# Pott's Paraplegia in a 2 Years Female: A Rare Presentation at an Early Age

**DOI:** 10.1155/2024/5575592

**Published:** 2024-04-27

**Authors:** Preeti Basnet, Anish Joshi, Saurab Karki, Anil Jung Thapa, Prayash Poudel, Anugya Sapkota, Manoj Shrestha, Shreebridhi Pande

**Affiliations:** ^1^Kathmandu University School of Medical Sciences (KUSMS), Dhulikhel, Nepal; ^2^Nepalese Army Institute of Health Sciences (NAIHS), Kathmandu, Nepal; ^3^Kathmandu Medical College (KMC), Kathmandu, Nepal; ^4^Nepal Cardio Diabetes and Thyroid Center, Kathmandu, Nepal

## Abstract

**Introduction:**

Potts disease is extrapulmonary skeletal tuberculosis mostly affecting the thoracolumbar spine. It destroys the disc space, adjacent vertebral bodies, and spinal elements, leading to cord compression and paraplegia.

**Methods:**

This is a case report study of a 29-month-old toddler who presented to our hospital with bilateral lower limb weakness.

**Results:**

On clinical, laboratory, and radiological examination, she was diagnosed with Pott's spine, started on antitubercular therapy, and planned for surgery in her follow-up.

**Conclusion:**

Tuberculosis of the spine is still prevalent in developing countries, mainly in children. Complications of the disease can be devastating because of its ability to cause bone destruction, spinal deformity, and paraplegia. So, in a tuberculosis-endemic region, clinical suspicion should be there for Potts disease when a child presents with paraplegia of the lower limbs. Children can develop tuberculosis which can spread to the spine despite vaccination. The prognosis of spinal tuberculosis is improved by early diagnosis and rapid intervention.

## 1. Introduction

Tuberculosis (TB) is a bacterial disease caused by the infection of Mycobacterium tuberculosis, which mostly affects the lungs. Extrapulmonary involvement includes the central nervous system, pleural, miliary/disseminated, lymphadenopathy, skeletal, and joint tuberculosis. Pott's spine, the most common cause of skeletal TB, mostly affects the lower thoracic and lumbar regions [1]. It usually destroys the disc space, adjacent vertebral bodies, and other spinal elements, progressively leading to kyphosis. The paraplegia resulting from it is termed Pott's paraplegia. Even after all diagnostic procedures, surgical techniques, and effective tuberculostatic drugs, Pott's disease is a serious, life-threatening condition whose diagnostic delay can lead to spinal deformity and spinal cord compression with neurological deficits [2].

Herein, we report a case of a 29-month-old toddler with complaints of bilateral lower limb weakness, swelling over the thoracic region, and cough. Contrast Enhanced Computed Tomography (CECT) of the thoracic region revealed vertebral fragmentation causing anterior angulation and wedging with epidural collection and collection in the anterior paravertebral region, while the Mantoux test was significant. The patient responded to anti-tubercular therapy and is being planned for surgical intervention for neurological deficits and swelling. The case report also intends to show that tuberculosis should always be considered in a tubercular endemic region when a child presents with lower limb weakness despite BCG vaccination. Early diagnosis and rapid intervention should be started for a better prognosis. This case report has been reported in line with the CARE guidelines [3].

## 2. Case Report

### 2.1. Patient Presentation and History

A 29-month-old toddler was brought to our hospital by her parents with complaints of coughing and bilateral lower limb weakness. The weakness was insidious and gradually progressing. At 15 months of age, this child who was walking normally was now neither able to stand nor walk even with support. However, there was no weakness in the upper limbs. There was no trauma, fever, or any history of loose motion. There was also no history of abnormal body movement, excessive crying, or urinary or stool incontinence. She also had a cough which was dry in character and associated with post-tussive vomiting. On further investigation, her parents revealed she had lost her weight with loss of appetite. There was no quantification of the loss of weight. Her birth history was not significant. The child was vaccinated as per the national immunization schedule including a BCG vaccine and came from a low socioeconomic background. Her past medical history was significant for pneumonia at twelve months of age, where she received treatment with no sequelae.

### 2.2. Physical Examination

At presentation, the patient was ill-looking with a weight of 7.5 kg, height of 74 cm, and head circumference of 43 cm. Vital signs were stable. She had pallor and persistent anterior fontanelle which was flat and measured 1 × 1.5 cm. There was a swelling of size 6 cm × 7 cm on the dorsal thoracic spine, T4-T8 level, hard, non-mobile, non-tender, with no rise in temperature and no pus point. She had a pectus carinatum deformity with bilateral basal crepitations. The bilateral lower limb was hypertonic with decreased power, exaggerated reflex, clasp knife rigidity, and clonus which was more prominent towards the right side. The upper limbs were grossly normal, and the rest of her neurological examination was unremarkable.

### 2.3. Investigations

**Figure 1:**
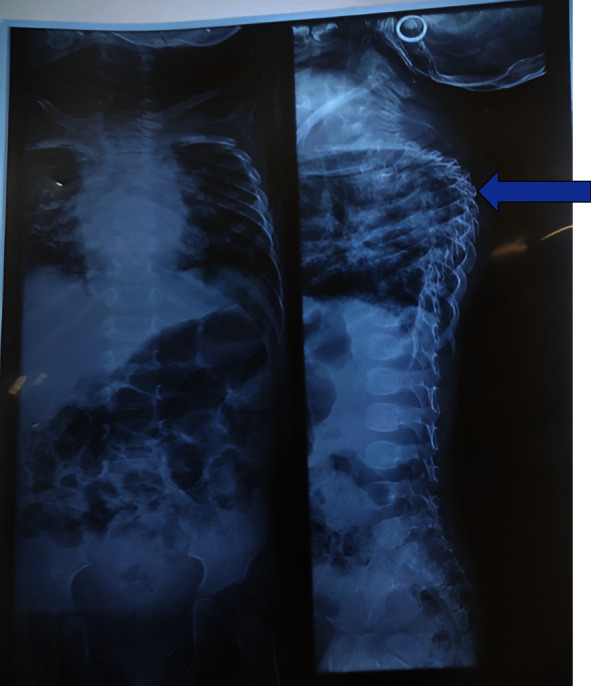
Chest X-ray showing diffuse infiltrates over the bilateral lung field and an X-ray of the spine showing kyphotic deformity of the thoracic vertebra (arrow).

Her investigations revealed leukocytosis (total WBC count of 14100 with 55% neutrophils and 35% lymphocytes) and her hemoglobin level was 9 g/dL. Liver, thyroid, and renal function were normal. The sputum sample showed normal respiratory flora. Chest X-ray revealed diffuse infiltrates over bilateral lung fields and an X-ray of the spine showed kyphotic deformity of the thoracic spine as shown in Figure 1.

**Figure 2:**
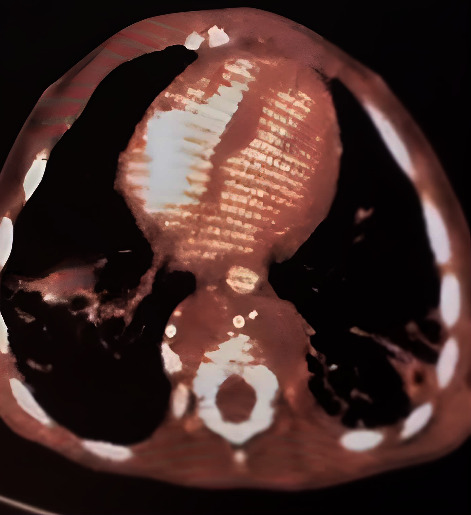
Contrast-enhanced CT axial view, showing cortical irregularity and erosion of the anterior aspect of the body of the thoracic vertebra with a dispersion of the bony fragments in the paraspinal region. There is evidence of paraspinal and prevertebral enhancing hypodense collection, suggesting a paraspinal collection.

Contrast enhanced computed tomography (CECT) revealed fragmentation causing anterior angulation and wedging with epidural collection and collection in the anterior paravertebral region with minimal enhancing wall with calcification as shown in Figure 2.

Her ESR was raised i.e., 55 mm/hour. A GeneXpert of the sputum was done which was negative for Mycobacterium. Mantoux test showed 10 mm of induration in 48 hours.

### 2.4. Treatment History

She was on IV antibiotics (Ceftriaxone and Amikacin) for a few days but her symptoms were not improving. Since there is the involvement of more than two vertebral levels with the paraspinal collection, raised ESR, with significant tuberculin skin test, and from tuberculosis-endemic region, she was started on antitubercular therapy (ATT) with isoniazid (50 mg)/rifampicin (75 mg)/pyrazinamide (150 mg) 2 tablets and ethambutol (100 mg) single tablet with pyridoxine 10 mg single dose every day. She was also given the syrup prednisolone (@2 mg/kg/day).

After 20 days of admission, her respiratory symptoms subsided but lower limb weakness was still persistent. She was discharged on ATT intensive therapy for 2 months with a plan to undergo surgical intervention on her follow-up.

## 3. Discussion

Potts disease is a tubercular disease of the spinal column. It was first described by Sir Percival Pott in patients presenting clinically with kyphotic deformity and neurological deficit [4]. Spinal tuberculosis is more common in children and younger adults in endemic countries, while the disease affects the adult population in developed Western and Middle East countries [5]. Approximately 10% of patients with extrapulmonary tuberculosis have skeletal involvement, among which half of them have spinal involvement followed by the hip and knee [1]. A retrospective study done in the UK revealed that the thoracic spine was the most affected region [5]. The main route of infection of spinal TB is through hematogenous spread from the primary infection site, which in most cases is unknown [6]. Mycobacterium is deposited in the vertebral body next to the anterior portion of the vertebral endplate through the terminating arterioles. Consequently, the most frequently affected region of the vertebral body is the anterior part of the vertebral end plate. While the infection is spreading, the cortex is disrupted, and anterior and posterior longitudinal ligaments along with the periosteum are destroyed [7]. The extension of infection into the nearby soft tissue can develop paravertebral or epidural masses leading to spinal cord compression and other neurological issues [6]. In children, the destruction of the vertebral column is more severe because most of the bone is cartilaginous and the angulation is mostly significant due to growth retardation of the anterior column and unrestricted growth of the posterior column [8].

The clinical features vary and may present with local pain, local tenderness, stiffness and spasms of muscles, cold abscess, or prominent spinal deformity [9]. The appearance of kyphotic deformity has been classified with vertebral involvement, with the knuckle being one, gibbus being two, and rounded kyphus being more than three vertebral involvements respectively [10]. Apart from clinical features, helpful investigations are the tuberculin skin test (positive in 90%), Mycobacterium tuberculosis culture which is positive in 67%, and MRI which is abnormal in all cases [11]. Neurological deficits may be seen in up to 45% of patients with spinal TB [12]. The neurological symptoms may be subtle at first, but with time numbness, tingling in the extremities, paresthesia in belt-like distribution around the chest wall, and a sense of weakness with activity may progress to its severe form. Motor functions are always affected first and to a greater extent than sensory functions because of the location of the diseased spine which lies anterior to the cord, thus being nearer to the motor tracts [7, 13]. Paraplegia in Pott's spine is due to one of three causes, i.e., direct bony pressure, displacement of a portion of bone or pressure due to sequestrum, and pressure due to caseous material invading the spinal canal causing not only mechanical pressure but also tuberculous pachymeningitis [4]. A neurological deficit occurs depending on the compromised level; cervical TB causes weakness, pain, and numbness of both upper and lower extremities evolving to full-blown quadriplegia, while thoracic and lumbar involvement spares the upper extremity [1]. In our case, the patient presented with lower limb weakness while the sparing the upper limbs, swelling over the back, and cough suggesting thoracolumbar involvement.

The diagnosis of Potts disease can be done by clinical, laboratory, radiological, and bacteriological confirmation. Culture along with histopathological analysis of the cold abscess is the ideal investigation but is difficult due to cultural, social, and socioeconomic issues [14]. In this case, we established the diagnosis by clinical features, radiographical imaging of the patient, and tuberculin skin test to support our diagnosis. In a majority of the cases, the diagnosis of spinal tuberculosis was confirmed by radiological characteristic findings along with other positive findings such as a clinical history of fever and anorexia, positive tuberculin skin test, suggestive chest radiography or chest CT scan findings, and/or a positive response to antituberculous drug therapy [15].

The prognosis for spinal tuberculosis is improved by early diagnosis and rapid management [9]. In children, the source of infection for skeletal TB is hematogenous spread from the primary site. The time of bone infection may vary from a month to years after primary infection, and the onset of symptoms usually takes 1–3 years after infection [16]. In a study conducted by Gupta et al. 50 patients with Pott's spine were studied, which included 8 children. Among all patients, paraplegia was only found in 5 of them, which accounts for only 10% of the study population [17]. However, data on the pediatric population with paraplegia is lacking and more research is needed for knowing about the incidence of Pott's paraplegia in children. Similarly, the article by Bastola et al. mentions that it requires at least one year to have the onset of symptoms of osteoarticular tuberculosis from the time of primary infection and the mean age at diagnosis is 1.9 years [18]. Hence, it is extremely rare to have a severe form of disease very early in this age group.

In a meta-analysis done by Martinez et al. and his team—Infant BCG vaccination and risk of pulmonary and extrapulmonary tuberculosis throughout the life course, 1782 (26%) developed tuberculosis of which 1309 were BCG vaccinated and 473 were unvaccinated among 68,552 participants. The same study showed the overall effectiveness of BCG vaccination against all tuberculosis was 18% and when stratified by age, vaccination only significantly protected against all TB in children younger than 5 years [19]. So, despite receiving BCG vaccination, our patient developed Pott's paraplegia at a very young age.

The limitations of your case report on Pott's disease in a pediatric patient include its single-case nature, which makes generalization challenging, and the fact that it originates from a developing region with a higher prevalence of tuberculosis (TB), potentially limiting its relevance or generalizability to developed regions where TB may not be as prevalent.

## 4. Conclusion

Tuberculosis of the spine is still prevalent in developing countries, mainly in children. Complications of the disease can be devastating because of its ability to cause bone destruction, spinal deformity, and paraplegia. So, in a tuberculosis-endemic region, clinical suspicion should be there for Potts disease when a child presents with paraplegia of the lower limbs. Children can develop tuberculosis which can spread to the spine despite BCG vaccination. The prognosis of spinal tuberculosis is improved by early diagnosis and rapid intervention.

## Data Availability

The data used to support the findings of this study are included in the article.
